# A prevalence of running-related injuries among professional endurance runners in the Rift Valley, Kenya

**DOI:** 10.17159/2078-516X/2021/v33i1a10690

**Published:** 2021-09-23

**Authors:** RC Koech, B Olivier, N Tawa

**Affiliations:** 1 Department of Physiotherapy, Jomo Kenyatta University of Agriculture and Technology, Kenya; 2 Centre for Research in Spinal Health and Rehabilitation Medicine, Department of Physiotherapy, Jomo Kenyatta University of Agriculture and Technology, Kenya; 3 Department of Physiotherapy, Faculty of Health Sciences, University of the Witwatersrand, Johannesburg, South Africa

**Keywords:** occurrence, injuries related to running, experienced runners, Kenya

## Abstract

**Background:**

Injuries related to endurance running have attracted attention as the sport has become more competitive, and as athletes seek to improve their performance. Consequently, endurance runners are increasingly becoming more susceptible to lower extremity running injuries.

**Objectives:**

The aim of this study was to establish the prevalence of running-related injuries among professional endurance runners in the Rift Valley, Kenya.

**Methods:**

We used a cross-sectional survey design targeting professional endurance runners who had participated in both local and international running competitions. The sample size consisted of 209 respondents selected through stratified and simple random sampling techniques, of which 167 participated in the study. A self-administered questionnaire was used to collect data on the prevalence of injuries among the endurance runners. The data were analysed using descriptive statistics.

**Results:**

The prevalence of running injuries was 63% (n=106). The prevalence among males (n=64; 69%) was higher in comparison with that of females (n=42; 57%). The posterior thigh was the most common site for injuries among the athletes (n=87; 52%), followed by the lower back (n=78; 47%) and ankle (n=63; 38%).

**Conclusion:**

The prevalence of running-related injuries was high among professional Kenyan endurance runners compared to other populations. These findings therefore form the basis of future research to explore the mechanisms behind the injuries and the feasibility of targeted injury prevention programmes.

Endurance or long-distance running is a continuous run on the track or off the track over distances ranging from eight hundred metres extending to full marathons and ultra-marathons.^[1]^ Kenyan endurance runners have featured prominently in races organised by the World Cross-Country Championships (WCCC) and the International Association of Athletics Federations (IAAF). ^[2]^ According to Fisher, nearly 70% to 80% of endurance running winners in any global event since the late 1980s are of Kenyan descent. ^[3]^

The issue of injuries among runners has been a concern to various stakeholders, including researchers. As a result, a growing number of studies have been published recently with the aim of addressing this problem. For instance, a systematic literature review on predisposing factors, incidence and prevention of injuries in endurance running by Videbæk et al. ^[4]^ revealed that injury prevalence rates among recreational and novice distance runners were higher than those of marathoners. The differences in incidence rates were significant in competitive runners, i.e. marathoners, having injury incidence rates as low as 2.5 to 7.4 per 1 000 hours while non-competitive runners, such as novice runners, registered injury incidence rates of up to 17.8 injuries per 1 000 hours of exercise. ^[4]^ Small and Relph ^[5]^ showed that 26 runners sustained 108 lower extremity injuries (89%) in a multiday marathon lasting 27 days, indicating an average of four injuries per marathon runner. However, the study suggested that further investigation of the prevalence and predisposing factors using a larger sample size is required to allow proper generalisation of results.

Vitez et al. ^[6]^ investigated running-related injuries in runners of the Ljubljana Marathon in Slovenia and reported that a third of these marathon runners suffered at least one mild running injury during the season, with a 53% lifetime of running injury prevalence. The predominant injury region was the knee (30%). Iliotibial band syndrome, Achilles tendonitis and tibial stress syndrome were among the most common injuries reported by endurance runners (Tonoli et al. ^[7]^). Mbarak et al. ^[8]^ studied running-related musculoskeletal injuries, risk factors and treatment among short-, medium- and long-distance Kenyan runners pointing out the frequency of injuries at the injury sites. Hamstring injuries were the commonest (43/108 cases; 40%), followed by knee injuries (33/108 cases; 31%). However, the study did not specifically indicate the overall prevalence among the runners.

Therefore no study has focused specifically on the prevalence of running-related injuries among endurance runners in Kenya. Furthermore, most studies have been carried out in other contexts and have seldom focused on professional endurance runners of international stature. Kenya has a unique context for running injuries and local findings can be used to better inform injury prevention approaches and ultimately benefit running performance. This study therefore sought to establish the injury prevalence among professional endurance runners in the Rift Valley, Kenya.

## Methods

This cross-sectional survey was conducted in the Rift Valley region of Kenya. Based on registration data from Athletics Kenya (Athletics Governing Body), the study targeted 2 481 endurance runners who had participated in both local and international running competitions. Calculations using a proposed formula by Cochran ^[9]^ gave a sample size of 209 professional endurance runners. The study used stratified random sampling to distribute the sample size based on gender (male, n=119; female, n=90). A simple random sampling technique was then used to select the study’s participants, which meant that they had to have participated in one official local or international competition. Moreover, this population was chosen because of their availability as most of the runners were resident and training within the Athletics Kenya approved and recognised training camps. Runners below age of 18 years, running races less than 800 metres in length, and amateurs and foreigners were excluded from participating in the study.

The study used a pretested, self-administered questionnaire to collect the data. A running injury was defined as any muscle, bone or joint injury of the lower extremity and back (i.e. foot, ankle, calf, shin, knee, thigh, hip, lumbar region) resulting from participating in a training and/or competitive sports activity resulting in the runner missing out for >48 hours. An acute injury was defined as an injury caused by a particular and recognizable traumatic incident experienced by the athlete during training or competition whereas overuse injury was defined as having occurred as a result of recurrent microtrauma that could not be traced to a particular event or incident. To ascertain validity, the questionnaire was reviewed by two sports physiotherapists and twenty endurance runners prior to being administered to the respondents. The questionnaires administered to the runners had to be returned within two weeks. The researcher was available via mobile phone and at the training camps to clarify uncertainties where needed, while the respondents were completing the questionnaires. The data collection process took place over a period of 16 weeks (July-October) in 2019.

The data were processed using the Statistical Package for Social Sciences (SPSS version 22.0) and analysed using descriptive statistics. Permission to conduct the study was given by the relevant research regulatory bodies, and the endurance runners voluntarily consented to participate in the study after being assured that the data collected will be safe, secure and confidential.

## Results

### The demographic characteristics of the runners

A total of 182 questionnaires were returned. Fifteen of these were incomplete and thus could not be used for the study, leading to a final data analysis on 167 questionnaires. The demographic characteristics are presented in Table 1. The findings indicate that the majority of the respondents were male (n=93; 56%); aged 26–30 years (n= 63; 38%); had running experience of between 1–3 years (n=84; 50%); participated in both local and international competitions (n=62; 37%); the highest level of education was secondary level (n=120; 72%); and they depended on running as an occupation (n=127; 76%).

### The injury prevalence of running injuries

The responses on running-related injuries sustained within the previous year are shown in Table 2. The majority (n=106; 64%) of the athletes had experienced a running-related injury at some point within the previous year. The prevalence of running injuries was higher among males (n=64/93; 69%) than females (n=42/74; 57%). An overuse injury (n=74/106; 70%) was the most common type of injury among the runners.

The athletes were further asked to indicate the number of times they had experienced running-related injuries in their different body parts in the previous year while training or during running competitions. These results are given in Table 3. Posterior thigh injuries (n=87; 52%) were most common among the athletes, followed by lower back pain (n=78; 47%) at least once in the previous year while training or during running competitions. The third and fourth most prevalent injuries sustained during training and competition, respectively, were ankle (n=63; 38%) and groin (n=49; 29%) injuries.

## Discussion

Prevalence of injuries related to endurance running was n=106 (64%) which were incurred during the previous season. The majority of those injured suffered overuse injuries (n=74; 70%) possibly as a result of the combined effect of long-lasting fatigue beyond their specific biomechanical capability.^[10]^ In addition, the majority of the runners might have been predisposed to running injuries due to the observed overtraining behaviour aimed at coping with the competitiveness of long-distance running in Kenya.^[8]^ The injury rates reported among the athletes were higher than those of Vitez et al.^[6]^ in Slovenia who reported that a third of the Ljubljana marathoners experienced at least one mild running injury in the season. This is considered to be so because of the low sensitivity by the use of >2 weeks’ time-loss based on the injury definition by Vitez et al. Similarly, the injury prevalence among the endurance runners in this study was lower (n=167; 63%) than those found by Ellapen et al. ^[11]^ in KwaZulu-Natal in South Africa who observed that within a year, 90% of the 200 half marathon recreational runners suffered at least one running injury. Whereas the current study analysed data on 167 professional endurance runners and defined ‘injury’ as an occurrence happening during training and/or competition which made a runner lose more than 48 hours. Ellapen et al. dealt with 200 half marathon runners and classified an ‘injury’ as an incident that caused more than 24 hours of time lost, thus explaining the variation between the two studies. According to Tonoli et al. ^[7]^ experienced runners tend to incur fewer injuries because there is the development of an innate ability to recognise the onset of an injury thus preventing its severity compared to novice runners. This concurs with the result in Table 1 that the majority of the endurance runners (n=84; 50%) had professional running experience of between one to three years.

More males (n=64; 69%) than females (n=42; 57%) reported experiencing an injury within the 12-month period of our study. Since the external training conditions were the same for both females and males, this finding implies that the variation of injuries across gender lines is possibly due to internal factors i.e. anatomical, biomechanical and/or physiological.^[12]^ The difference in injury prevalence across both genders are similar to, although much higher than those of van der Worp et al. ^[13]^ in the Netherlands who found that male runners were more susceptible to calf and hamstring injuries, whereas women were more prone to sustaining hip injuries. It can therefore be concluded that men were at greater risk than women for developing running-related injuries.

Running-related posterior thigh injuries (n=87; 52%) was the most common injury site in both men and women, followed by the lower back (n=78; 47%). The running surface in the Rift Valley region is characterised by hills, valleys and flat terrain. Therefore, running in this region predisposes a runner to excessive loading of the quadriceps muscles (uphill training) resulting in an agonist and antagonist muscle imbalance with a higher prevalence of posterior thigh injuries. ^[14]^ It can be further suggested that high posterior thigh injuries were as a result of poor running methodology seen amongst the Kenya runners that involved inadequate warm-up, limited stretches and eccentric strength training. ^[8]^ The results in this study on the prevalence of posterior thigh injuries were, however, much higher than those of Opar et al. ^[15]^ who found that posterior thigh injuries accounted for 24% of the overall of the lower extremity running-related injuries. The comparatively high prevalence of injuries in this study compared to those of Opar et al. could be attributed to the fact that their study was carried over a duration of three years, while this study was based on the respondent’s recollection of injuries for the previous year. The injury prevalence of the lower back region (n=78; 47%) indicates that the runners might have been more predisposed to this injury due to excessive biomechanical overload as a result of overtraining, leading to increased fatigue and stress on the intervertebral disc. ^[9]^

The other common injury site reported by the athletes was the ankle (n=63; 38%). Large amounts of high-intensity running, rapid increase in training mileage and/or variation of running surfaces seen among the runners could possibly be an injury risk for ankle injuries, specifically if running on a hard surface. ^[8]^ These findings were slightly higher than those of Tonoli et al. ^[7]^ who found the prevalence rate of ankle injury among marathoners to be 25%. However, the ankle injury prevalence in this study was much higher than the 17 % prevalence rate reported by Small and Relph ^[5]^ among multiday marathoners. The high prevalence observed in this study compared to that of Small and Relph, ^[5]^ could be attributed to the fact that our study focused on professional camp-based runners, while the previous study focused on recreational marathoners.

This study is the first to report on the epidemiology of injuries amongst Kenyan endurance runners and these findings would be useful in future research. A limitation of this study is that only 167 questionnaires were included in the analysis, while the calculated sample size was 209. Although the findings reported here still makes a valuable contribution to the literature, caution should be taken in generalising results to the wider endurance runner population.

## Conclusion

The prevalence of running-related injuries among professional Kenyan endurance runners was 63% in a previous season. This forms the basis of future research to explore mechanisms behind running injuries and the effectiveness of intervention programmes to prevent injury. Future studies could also explore the extreme dominant performance of Kenyan endurance runners despite the high injury prevalence reported.

## Figures and Tables

**Table 1:** The demographic characteristics of the runners (n=167)

Variable	Category	Frequency (n)	Percentage (%)
**Gender**	**Male**	93	56
	**Female**	74	44

**Age in years**	**16 – 20**	33	20
	**21 – 25**	50	30
	**26 – 30**	63	38
	**31 – 35**	18	11
	**36 – 40**	1	1
	**41 – 45**	1	1
	**46 – 50**	1	1

**Professional running experience in years**	**1 – 3**	84	50
	**4 – 6**	57	34
	**7 – 10**	26	16

**Level of competitions**	**Local**	54	32
	**International**	51	31
	**Both**	62	37
	**Primary**	22	13

**Level of education**	**Secondary**	120	72
	**Certificate**	12	7
	**Diploma**	7	4
	**Undergraduate**	5	3
	**Postgraduate**	1	1

**Occupation other than athletics**	**Yes**	40	24
	**No**	127	76

**Table 2:** Running-related injuries sustained and their classification during the previous year (n=167 partcipants)

		Have you experienced a running injury within the previous year?			Indicate the type of injury sustained within the previous year		

Gender		Yes	No	Total	Acute^a^	Overuse^b^	Total
**Female**	n	42	32	74	15	27	42
	%	57	43	100	36	64	100

**Male**	n	64	29	93	17	47	64
	%	69	31	100	27	73	100

**Total**	n	106	61	167	32	74	106
	%	63	37	100	30	70	100

a Acute injury is injury sustained from a particular event that can be identified by the athlete.

b Overuse injury results from recurring microtrauma that cannot be traced to a single event.

**Table 3:** Number of injuries per injury site in the previous year (n=167 participants)

Injury site		Participants without injury	Participants with injury	Four or more times	Three times	Twice	Once
**Lower back**	n	89	78	26	17	9	26
	%	53	47	33	22	12	33

**Buttocks**	n	126	41	10	3	7	21
	%	75	25	25	7	17	51

**Groin**	n	118	49	5	7	13	24
	%	71	29	10	14	27	49

**Hip**	n	121	46	8	9	13	16
	%	72	28	17	20	28	35

**Anterior thigh**	n	121	46	10	11	13	12
	%	72	28	22	24	28	26

**Posterior thigh**	n	80	87	21	13	19	34
	%	48	52	24	15	22	39

**Knee**	n	124	43	16	4	11	12
	%	74	26	37	9	26	28

**Upper leg**	n	125	42	12	8	11	11
	%	75	25	29	19	26	26

**Lower leg**	n	122	45	9	6	16	14
	%	73	27	20	13	36	31

**Ankle**	n	104	63	8	9	3	43
	%	62	38	13	14	5	68

**Foot**	n	140	27	3	5	8	11
	%	84	16	11	18	30	41

**Toes**	n	132	35	3	6	6	20
	%	79	21	9	17	17	57

Injuries include acute and overuse injuries.
